# Conceptual Knowledge Influences Decision Making Differently in Individuals with High or Low Cognitive Flexibility: An ERP Study

**DOI:** 10.1371/journal.pone.0158875

**Published:** 2016-08-01

**Authors:** Xiaofei Dong, Xiumin Du, Bing Qi

**Affiliations:** College of Education, Hebei University, Baoding, China; Centre national de la recherche scientifique, FRANCE

## Abstract

**Objective:**

Studies using the Iowa Gambling Task (IGT) have distinguished between good and bad decision makers and have provided an explanation for deficits in decision making. Previous studies have demonstrated a link between Wisconsin Card Sorting Test (WCST) performance and IGT performance, but the results were not consistent and failed to explain why WCST performance can predict IGT performance. The present study aimed to demonstrate that WCST performance can predict IGT performance and to identify the cognitive component of the WCST that affects IGT performance using event-related potentials (ERPs).

**Methods:**

In this study, 39 healthy subjects (5 subjects were excluded) were divided into a high group and a low group based on their global score on the WCST. A single-choice version of the IGT was used to eliminate the impact of retrieval strategies on the choice evaluation process and interference due to uncorrelated decks. Differences in the underlying neural mechanisms and explicit knowledge between the two groups during the three stages of the decision-making process were described.

**Results:**

Based on the information processing perspective, we divided the decision-making process into three stages: choice evaluation, response selection, and feedback processing. The behavioral results showed that the highly cognitively flexible participants performed better on the IGT and acquired more knowledge of the task. The ERP results showed that during the choice evaluation stage, the P300 recorded from central and parietal regions when a bad deck appeared was larger in the high group participants than in the low group participants. During the response selection stage, the effect of choice type was significant only in the frontal region in the high group, with a larger effect for passing. During the feedback evaluation stage, a larger FRN was evoked for a loss than for a win in the high group, whereas the FRN effect was absent in the low group.

**Conclusion:**

Compared with the participants with low cognitive flexibility, the participants with high cognitive flexibility performed better on the IGT, acquired more knowledge of the task, and displayed more obvious somatic markers. The low group participants showed reduced working memory abilities during the choice evaluation stage. The appropriate somatic markers reflected by the DPN is formed only when conceptual knowledge is gained in the response selection stage. The absence of an FRN effect in the subjects who performed poorly on the WCST suggests a significant deficit in feedback learning and reward prediction.

## Introduction

### The IGT and Ambiguous Decision Making

Decision making is a process of assessing options and choices [1]. It involves inference and the processing of emotions and may be rational or irrational. Completely rational decisions do not exist in real life; most of the time, people rely on intuition and heuristics. The process through which people weigh pros and cons based on knowledge of probabilities and outcomes is called risky decision making. When the knowledge of probabilities and outcomes is insufficient, this process becomes ambiguous decision making [2].

In the Iowa Gambling Task (IGT) [3], individuals learn a reward-punishment rule over multiple trials. The IGT is a task of rule learning that includes both ambiguous decision making and risky decision making [4]. In this task, participants are asked to choose one of four decks. Each deck has rewards and punishments. Decks A and B are disadvantageous decks; they have large rewards, but they also have large punishments and end in loss over the long term. Decks C and D are advantageous decks; they have small rewards and punishments, and participants can earn money over the long term.

Studies that have used the IGT have distinguished between good and bad decision makers and have provided an explanation for deficits in decision making. According to the somatic marker hypothesis, emotional markers can be used as guides to explain participants’ performance on the IGT [5]. This hypothesis claimed that good decision maker have different somatic signals to favorable decks and unfavorable decks. Studies found that the anticipatory skin conductance recordings (aSCR), a typical somatic signals, is different between the advantageous and disadvantageous decks which provided support for the somatic marker hypothesis. And some researchers claimed that this somatic marker was present before the participants were explicitly aware of the rules. However, other researchers have emphasized the importance of cognitive abilities and conceptual knowledge. Cognitive flexibility is one of the cognitive abilities and attract much attentions which can be measured by the Wisconsin Card Sorting Test (WCST). Cognitive flexibility including working memory, abstract generalization, information extraction, categorization and implicit learning. Many studies have demonstrated that cognitive flexibility and IGT performance are related, but such studies have rarely explained the mechanistic connection between cognitive flexibility and decision making [6–8].

### The WCST and IGT

Direct support for the relationship between the IGT and WCST has been provided by psychiatric studies, but the results have not been conclusive. Some studies have found no significant relationship between performance on the WCST and IGT. For example, Ritter found that IGT performance was not correlated with WCST performance in schizophrenia patients [9], and Starcke obtained the same result for obsessive compulsive disorder (OCD) patients [10]. However, other studies have obtained the opposite results. For example, Yip found that subjects with schizophrenia performed significantly worse than non-psychiatric controls on both the IGT and the WCST [6] and that performance on these tasks was significantly correlated across both subject groups. The inconsistent results of these studies may be because the subjects of the studies were psychiatric patients with unstable cognitive abilities.

Nevertheless, studies that have focused on healthy samples have produced conclusive results. Lehto and Elorinne found that children’s perseverative responses on the WCST correlated significantly with performance on the IGT [7]. Brand also found a significant correlation between WCST and IGT performance (after one block) among healthy samples [8]; although the rule was initially random, after one block, these subjects had learned the rule by using their strategic and cognitive abilities. Delpero showed that the low performance of older people on the IGT and WCST [11] was due to their lower sensitivity to feedback, as measured by the Pictures Decision Task. These relational studies have provided evidence that WCST performance is a good predictor of IGT performance. However, the theoretical explanations remained to be determined.

No research has focused on the neural mechanisms of WCST and IGT performance simultaneously, but some studies suggest that the WCST recruits some of the same cognitive components as the IGT. Many studies have indicated that WCST performance is associated with activity in the dorsolateral prefrontal cortex (DLPFC) [12], and cognitive flexibility, which is measured by the WCST, is regarded as the basis of decision making [13]. Kaladjian et al. suggested that execution of the WCST and the IGT depends on joint contributions of the DLPFC [14], orbitofrontal cortex (OFC) and ventromedial prefrontal cortex (vmPFC). Damage to the DLPFC can indirectly impact vmPFC functioning [15], and damage to the OFC can lead to inhibitory deficits as well as more perseverative errors on the WCST, although this task is considered to reflect DLPFC functioning [16]. Thus, OFC, vmPFC, and DLPFC functioning may all be essential for valid executive functioning and rational decision making. It can thus be inferred that low performance on the WCST predicts deficits in decision making.

The neural mechanisms that explain why the WCST can predict decision making performance can be investigated over the course of decision making using electroencephalography (EEG). Event-related potential (ERP) studies of the IGT have clearly demonstrated the process of decision making and have provided an explanation for why some people can make advantageous decisions whereas others cannot. According to the information processing perspective, the decision-making process can be divided into three stages: choice evaluation, response selection and feedback processing. The P300, decision preceding negativity (DPN) and feedback-related negativity (FRN) have been analyzed as correlates of these three stages, [17]. The P300 is a positive-going component that peaks between approximately 250 and 500 ms after stimulus onset and originates from central and parietal regions. The P300 appears during the early stage of processing, reflects stimulus discrimination and is a sign of attention and working memory [18]. The DPN is a slow, negative potential that is recorded over the right anterior regions and precedes choice selection by approximately 500 ms [19]. This component reflects anticipation for the decks. The FRN is a negativity that originates in the medial frontal cortex [20] and that occurs approximately 265 ms after feedback for a win or loss. The FRN reflects the process of implicit learning through feedback. A summary of the cognitive processes that have been proposed to be at work in the IGT and the ERP components used to evaluate them is presented in Table 1.

**Table 1:** Summary of cognitive processes involved in the IGT and the corresponding ERP components.

Stage of IGT	Cognitive processes in the IGT	ERP components	References
Choice evaluation	Working memory; attention; perception of stimulus	P300	Horowitz-Kraus, 2014;Maurage et al., 2007
Response selection	anticipation of risky choices; appreciation of the consequences of choices; intuitive judgments	DPN	Cui et al., 2013; Bianchin et al., 2011; Giustiniani et al., 2015
Feedback evaluation	learning from feedback; appraisal of the benefit of outcomes	FRN	Cui et al., 2013; Balconi et al., 2015; Toyomaki et al., 2004; Hajcak et al., 2005

Some studies of the IGT have helped provide a theoretical explanation for the relationship between the WCST and IGT. But there is a debate about the role of cognitive processes and somatic markers in IGT performance. The continual debate may be because researchers have tended to focus only on one aspect or the other. Some studies have considered conceptual knowledge to be essential in the decision making process—even more important than somatic markers [21,22]. Maia and McClelland noted the possibility that both conceptual and unconscious knowledge is employed in the IGT [23]. Hinson et al. noted that working memory can affect the formation of aSCRs [24], and Jameson et al. found that interference with the central executive could damage the development of somatic markers [25]. The goal of the present study was to investigate how somatic markers affected by cognitive ability through the neural basis and conceptual knowledge of the IGT.

### The Aim of the Current Study

Some aspects of previous studies need to be improved. First, previous studies have largely focused on psychiatric subjects, and studies of these subjects cannot provide a cognitive mechanism to explain why WCST performance is a good predictor of IGT performance. The primary goal of these studies is typically to describe the patients’ psychiatric symptoms, of which impaired decision making is only one. Differences in the performance of healthy individuals with high and low cognitive ability need to be investigated. Second, few studies have simultaneously examined how WCST performance affects IGT performance, the underlying neural mechanisms, and the importance of explicit knowledge. It is necessary to specifically explore how highly cognitively flexible individuals act in the IGT from both the implicit learning and explicit knowledge perspectives. For instance, it is important to examine which aspects of cognitive flexibility (e.g., attention, updating of task-set information in working memory, anticipation of decks, feedback learning or explicit cognition) play a role in IGT performance.

By dividing healthy subjects into high and low groups based on their global score on the WCST, this study examined the relationship between WCST and IGT performance. The aim was to describe the differences between these two groups during the different stages of the decision-making process. A single-choice version of the IGT was used to eliminate the impact of retrieval strategies on the choice evaluation process and the interference caused by uncorrelated decks [26]. To examine the explicit understanding of the IGT, the participants were required to complete a simplified version of the questions utilized in Fernie’s study of explicit knowledge of the IGT after each block because both the WCST and IGT involve the learning of contingencies [21]. We expected that the learning pattern and explicit understanding of the IGT would differ between the high and low WCST groups and that the high WCST group participants would show better performance and consciousness of the rules of the IGT task.

This study aimed to identify the cognitive component of the WCST that affects IGT performance using ERPs. Based on previous ERP studies, we expected that for the choice evaluation stage, the low group would show a reduced P300 amplitude compared with the high group because working memory and attention affect the learning process. For the response selection stage, the expected result was the same as that found in Cui et al. [26]: the difference between “pass” and “play” was expected to be larger in the high group than in the low group. For disadvantageous decks, the high group subjects were also expected to show a larger DPN than the low group participants. For the feedback processing stage, the high and low WCST groups can be compared to examine whether feedback processing difficulties cause decision making deficits. We predicted that in line with previous studies, the FRN would be sensitive to the valence of the outcome. Therefore, we expected a loss to evoke a larger FRN than a win in the high group and that the low group would not show an FRN effect.

## Method

### Ethics Statement

The study was approved by the ethics committee of Hebei University. All patients provided written informed consent.

### Procedure

The experiment was introduced to the subjects, and their questions were answered. The WCST task was completed the day before the IGT was conducted. WCST performance was analyzed in terms of the global score and the number of perseverative errors. Based on the global score, the participants were divided into four levels: excellent, fine, medium and bad. The two middle groups were excluded. Then, before the IGT was conducted, the participants were provided with information about the recording of their answers. The participants were told that throughout the task, when each block ended, a deck rating from -10 to 10 (deck rating) would be recorded [21]. The subjects were informed before the experiment began that they would be paid according to their IGT performance. There was a break between each block. The EEG recording lasted 50–60 minutes, and the entire experiment, including preparation and debriefing, lasted 1.5–2 h.

### Participants

Thirty-nine right-handed students (mean age = 21.62, SD = 2.5, range = 19–26; education = 14.18 years) recruited from Hebei University participated in this study. Five subjects were excluded due to data recording problems. The rest of the subjects consisted of 17 high group participants (7 males, 10 females) and 17 low group participants (5 males, 12 females). All subjects had no history of neurological disorders, brain trauma, substance abuse, psychiatric diseases, alcoholic abuse or stroke. The subjects were informed of the purpose and process of the experiment and provided informed consent. The subjects were paid based on their final performance on the IGT.

### Stimuli and data acquisition

#### WCST

The computer version of the WCST modified by Heaton consists of 128 response cards and 4 stimulus cards [27]. Each card includes three dimensions of features: color (red, green, yellow, blue), shape (square, circle, pentagon, triangle), and number (one, two, three, four). The participant has to sort the cards according to their color, shape, or quantity of shapes. The rules change without notice, but feedback about each answer is given. A correct selection precedes a switch to another dimension. Thus, the subjects not only must learn and maintain in their working memory the correct matching rule while inhibiting irrelevant stimuli but also must exhibit cognitive flexibility in detecting when the rule has changed.

#### IGT Task

The modified version of the IGT was adopted from Cauffman et al. and Cui et al. to exclude irrelevant components from the response selection stage [26,28] and was adapted for ERP analysis and PC presentation. The frequency of wins in Deck A was 50%, with the magnitude of wins ranging from 50 to 250. The frequency of wins in Deck B was 90%, the magnitude of wins ranged from 80 to 130, and the magnitude of loss was -1150. The frequency of wins in Deck C was 50%, the magnitude of wins ranged from 40 to 70, and the magnitude of losses ranged from 5 to 25. The frequency of wins in Deck D was 90%, the magnitude of wins ranged from 40 to 70, and the magnitude of loss was -200. Decks C and D led to wins in the long run; the expected values were 200 for Deck C and 250 for Deck D. Decks A and B were disadvantageous; the expected value for Decks A and B was -250. With an initial funding of 2000 Yuan, the subjects had to earn as much money as possible.

A fixation point was initially presented in the middle of the screen for 1000 ms. A deck was emphasized with a yellow border, and the subjects were asked to choose to play or pass that card. During this choice evaluation stage, the subjects could not reply. Once the fixation point disappeared, the response selection stage began. The subjects were asked to choose to play or pass and could press “J” to play or “F” to pass. The feedback, which indicated the total amount of money and whether it was a win or loss, was presented on the screen for 1000 ms, and then the next trial was presented. The entire experiment contained 600 trials divided into 30 blocks. The subjects were told that the purpose of this task was to earn as much money as possible. The participants were required to complete a short version of the game including 20 trials to familiarize themselves with the decks. The entire task consisted of 600 trials, but the participants did not know how many trials they would complete.

To assess the participants’ knowledge of the game, questions were asked at 20-trial intervals (“On a scale of -10 to +10, how good or bad do you think deck A is, where -10 means that it is terrible and +10 means that it is excellent?” This question was repeated for Decks B through D). Based on the criterion used in previous studies [21,23], the participants in this study were divided into Level 0 and Level 1. If a participant was classified as Level 1, they would give the highest ratings to Decks C and D. If not, the participant was classified as Level 0. At Level 0, the subjects did not have the conceptual knowledge required to form a preference for one of the two best decks. At Level 1, the subjects had the conceptual knowledge needed to form a preference for the two best decks, but they did not have quantitative knowledge about the outcomes of the decks that could provide evidence for that preference. These two levels can also be called “pre-knowledge” and “post-knowledge”.

### EEG Recording and Analysis

Using Ag/AgCl electrodes, a sampling rate of 1000 Hz and a band-pass filter of 0–100 Hz, 32 scalp sites were used to record EEG data. The right mastoid was used as the reference for data recording, and the offline reference was the average of the left and right mastoids. The horizontal electrooculogram (EOG) electrodes were positioned at the outer canthi of the left and right eyes, and the vertical EOG electrodes were positioned above and below the left eye. Throughout the experiment, the impedance of all electrodes was maintained below 5kΩ. NeuroScan 4.3 was used to perform ocular reduction following offline digital filtering of the EEG data using a 30 Hz zero-phase-shift low-pass filter at 24 dB/oct. Trials in which the signal remained within ± 100 μV were retained; the others were artificially rejected.

Based on the decision-making processing stages, the EEG data were analyzed as follows.

#### Choice evaluation stage

Stimulus onset was set as time 0, and the epoch was from -200 ms to 1000 ms. The baseline was from -200 ms to 0. In this stage, the analysis time window for the P300 was 300–500 ms after stimulus onset. Nine electrodes (F3, FZ, F4, C3, CZ, C4, P3, PZ, and P4) were used for this analysis.

#### Response selection stage

Based on previous studies [19,26], the preliminary analysis used 600–800 ms after the choice as the baseline because the fluctuations were quite stable after the response. The analysis time window for the DPN was 800 ms before the response (we also referred to the average response time of 764 ms to set the time window). This study analyzed whether the DPN would differ based on choice (play or pass). The electrodes used for analysis were the same as those used in the choice evaluation stage.

#### Feedback evaluation stage

The main focus of this stage was how the valence of feedback affected the FRN. According to previous literature, such as Toyomaki and Murohashi’s study [29], the amplitude of the FRN is affected by the P200, and the amplitude of the FRN can be attained by subtracting the negative peak at 220–330 ms from the positive peak at 150–200 ms. Three electrodes (FZ, FCZ, and CZ) were used for this analysis. SPSS 15.0 was used to perform the analysis, and all analyses were Greenhouse-Geisser corrected.

## Results

### Behavioral Results

The results of a one-sample t-test showed that the high group won significantly more money than the low group, *t*_(32)_ = 2.147, *p* < 0.05. The mean of high group is 3353.24, SD = 1602.09; the mean of low group is 2367.06, SD = 1010.64. The data of this part is in S1 File.

**Fig 1:**
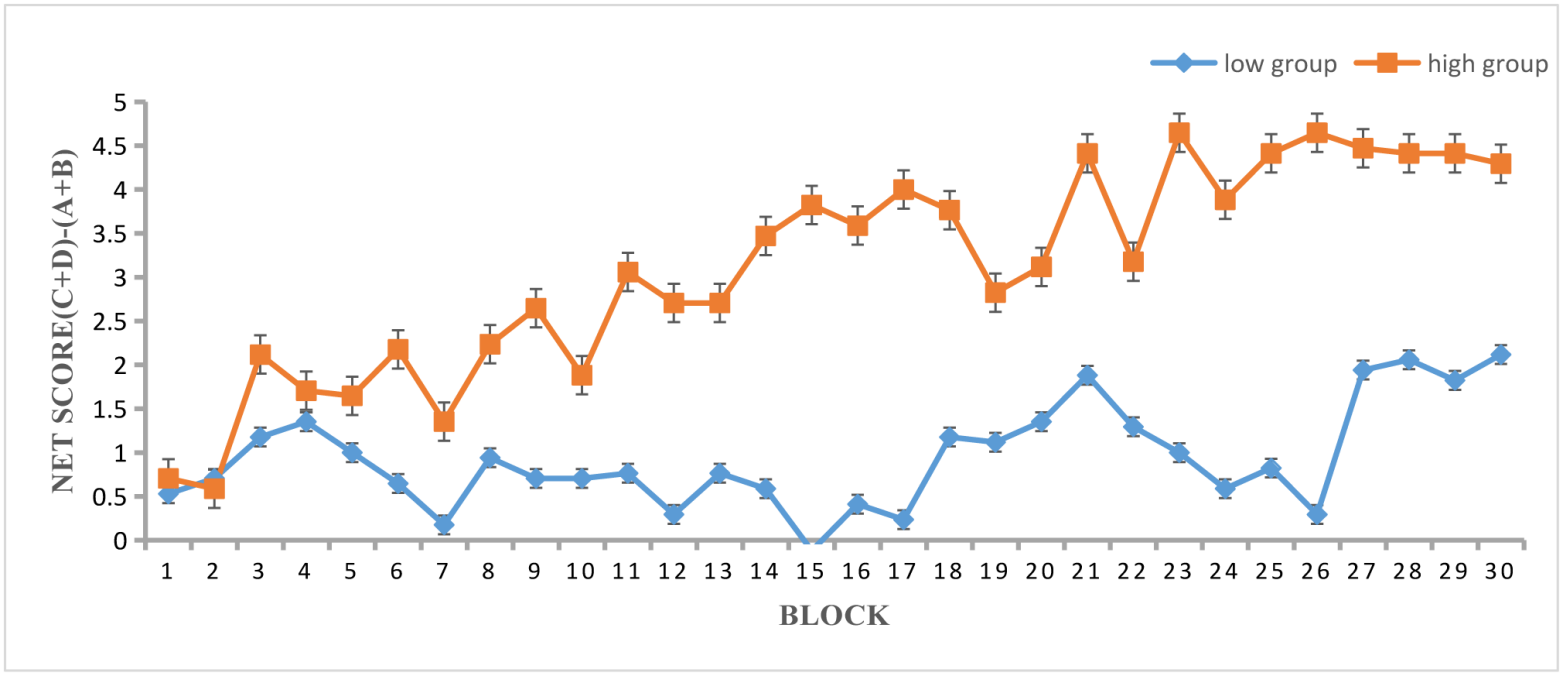
Mean net score for the high and low groups across blocks.

The mean net score was calculated using the formula (C + D)—(A + B). A preference for advantageous decks is evident if the score is positive, and an increasing mean net score can be used as a measure of the subjects’ learning process. The mean net score was calculated for each block and compared between the high and low WCST score groups across blocks 1–30 (see Fig 1). The result of a mixed-design ANOVA showed a significant main effect of group, *F*_(1, 32)_ = 11.755, *p* < 0.01, *η*^*2*^_*p*_ = 0.269. There was also a main effect of block, *F*_(29, 928)_ = 5.158, MSE = 11.663, *p* < 0.01, *η*^*2*^_*p*_ = 0.139, indicating that the mean net score increased as the number of trials completed increased. There was also an interaction, *F*_(29, 928)_ = 2.786, MSE = 11.663, *p* < 0.01, *η*^*2*^_*p*_ = 0.08, indicating that the high group learned at a faster pace than the low group.

### Knowledge of the Task: Deck Rating

The number of participants classified as Level 1 was calculated, and this number was then divided by the total number of participants in each group. The data of this part is in S2 File.

**Fig 2:**
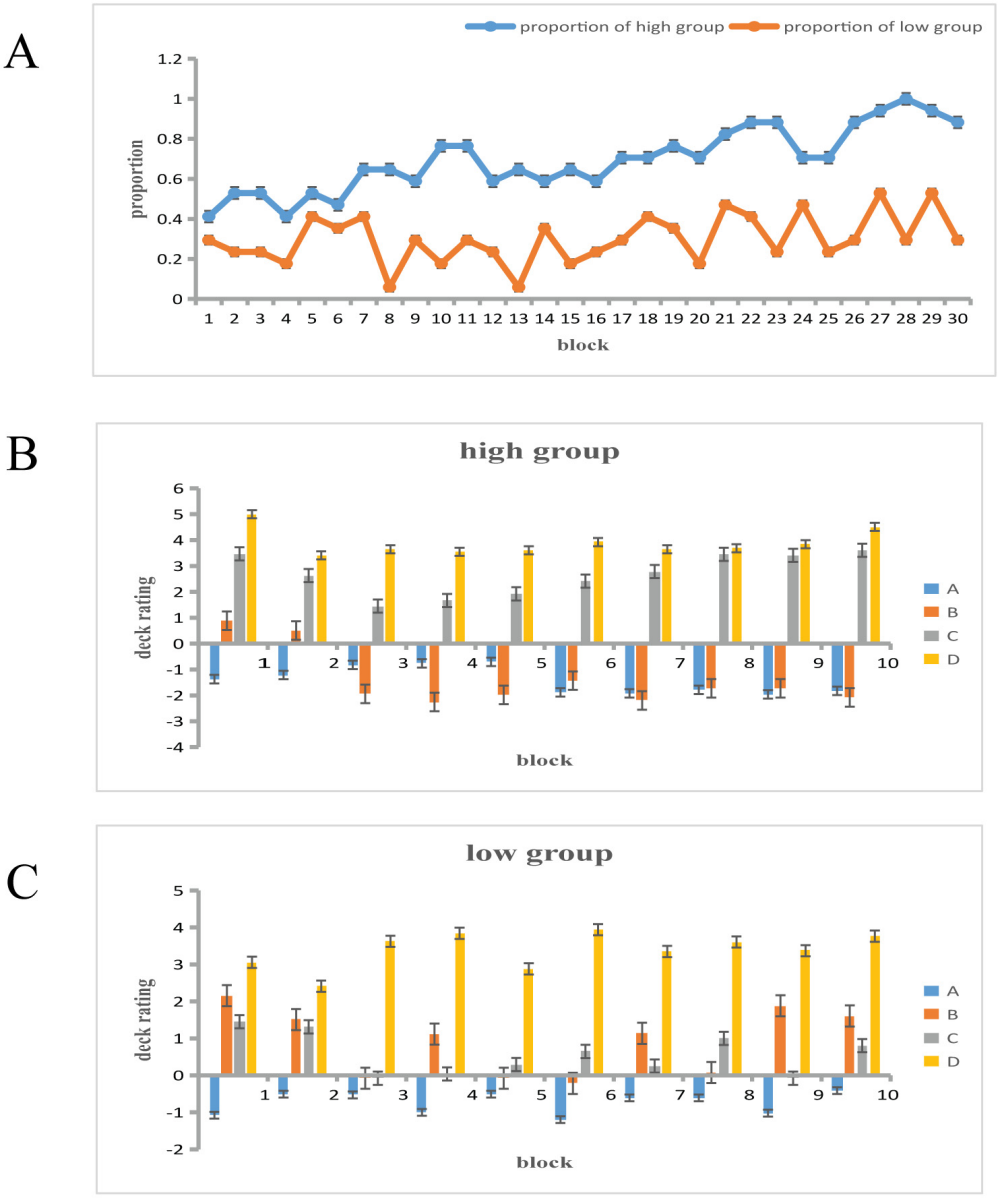
Knowledge of the task. (A) The proportion of participants who achieved conceptual knowledge between groups and across blocks. (B) The average deck rating reported every three blocks by the high group. (C)The average deck ratings reported every three blocks by the low group.

The proportion of the participants who obtained the conceptual knowledge state was calculated for each block and was then compared between groups and across blocks (see Fig 2(A)). A mixed-design ANOVA (Greenhouse-Geisser corrected) revealed a significant main effect of group, *F*_(1, 32)_ = 7.679, *p* < 0.01, *η*^*2*^_*p*_ = 0.194. There was also a main effect of block, *F*_(29, 928)_ = 2.191, MSE = 0.415, *p* < 0.05, *η*^*2*^_*p*_ = 0.064. There was no interaction, *F*_(29, 928)_ = 1.460, MSE = 0.415, *p* > 0.05, *η*^*2*^_*p*_ = 0.044. The results indicated that on average, 56.1% of the high group participants obtained the conceptual knowledge state (see Fig 2(B)), whereas the average proportion for the low group was 33.9% (**see** Fig 2(C)). This result shows the effect of the acquisition and application of conceptual knowledge on decision making.

To compare the ratings for the four decks between groups and across blocks, a mixed-design ANOVA (Greenhouse-Geisser corrected) was conducted. There was a main effect of block, *F*_(29, 3712)_ = 1.871, MSE = 24.491, *p* < 0.05, *η*^*2*^_*p*_ = 0.014. A significant main effect of deck was also found, *F*_(3, 128)_ = 16.04, MSE = 167.648, *p* < 0.01, *η*^*2*^_*p*_ = 0.273. A pairwise comparison analysis revealed that in the high group, Deck A was rated similarly to Deck B but significantly differently from Decks C and D, whereas in the low group, there was no difference between the ratings for the four decks.

### ERP Results

#### Choice Evaluation Stage

The ERP modulations were divided into two periods (pre-knowledge and post-knowledge) based on the mean net score and deck rating. This study used a strict criterion to define the post-knowledge state to ensure that the participants had full understanding of both advantageous decks, which Maia and McClelland called the “both” group [23]. If the participants did not return to Level 0 after reaching Level 1, then knowledge about the advantageous decks must be stable. The trials completed during the post-knowledge stage were analyzed for each participant, so the number of analyzed trials differed across participants (see Table 2). The data of this part is in S3 File.

**Table 2:** Participants’ knowledge stage across trials.

	n	Pre-knowledge	Post-knowledge
High group	1	1–200 trial	201–600 trial
	16	1–300 trial	301–600 trial
Low group	2	1–300 trial	301–600 trial
	15	1–450 trial	451–600 trial

**Fig 3:**
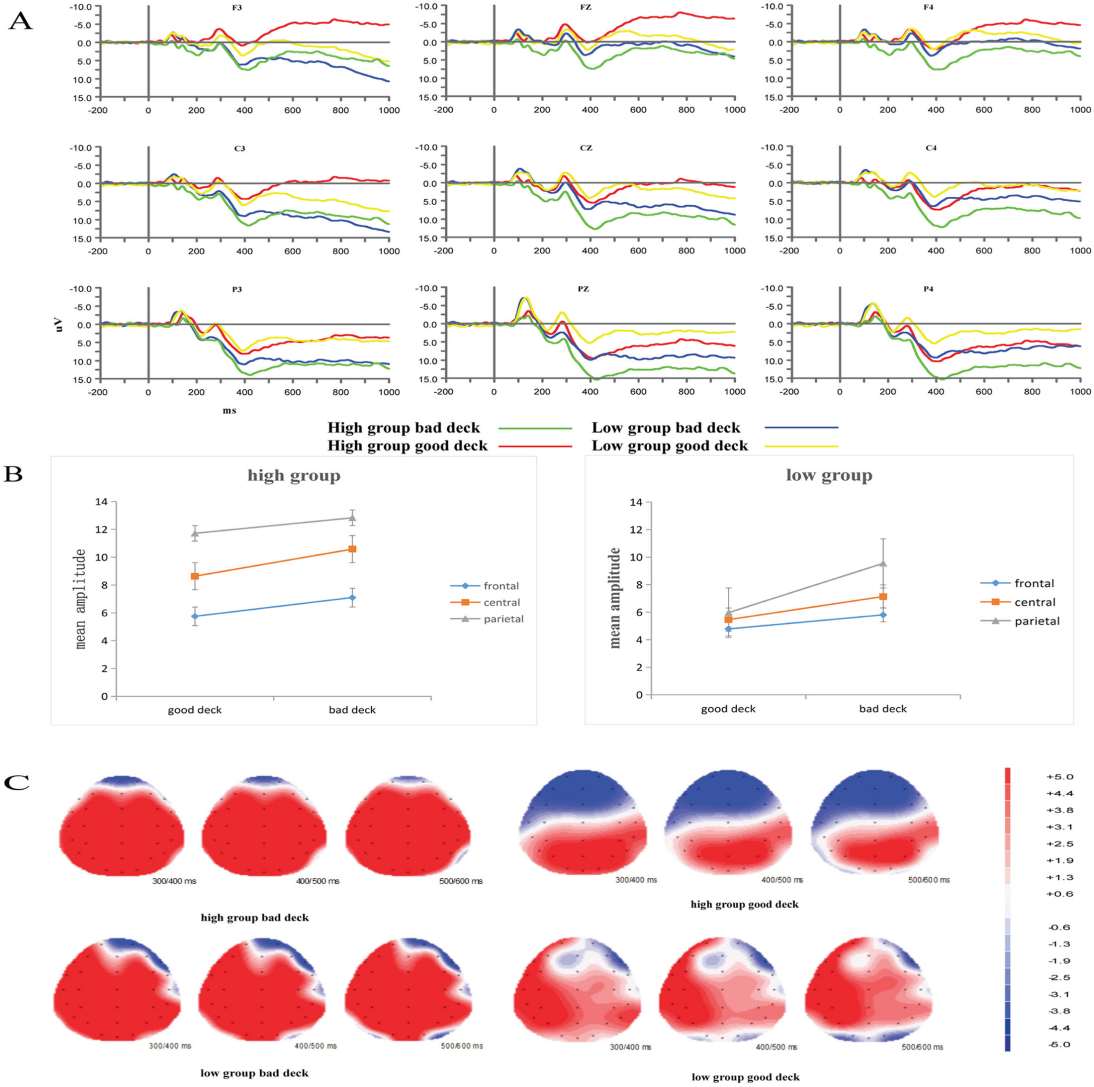
ERP results for the choice evaluation stage. (A) Grand average ERP wave forms after the onset of advantageous and disadvantageous decks for the two groups. (B) The mean amplitude of different regions for advantageous and disadvantageous decks in the two groups. (C) Topographical maps of the difference between disadvantageous and advantageous decks for the different wave forms in the two groups.

The mean amplitude of the P300 was calculated between 300–500 ms on 9 electrodes (F3, FZ, F4, C3, CZ, C4, P3, PZ, and P4). The wave forms of P300 of advantageous and disadvantageous decks for the two groups is presented in Fig 3(A). A 2 (group: high, low) × 2 (type of deck: advantageous, disadvantageous) × 3 (region: frontal, central, parietal) × 3 (laterality: left, middle, right) mixed-design ANOVA was performed. Group was the between-subjects factor, and the others were within-subject factors. A mean of 99 (SD = 5) and 115 (SD = 3.8) artifact-free trials were used in the analysis for the disadvantageous and advantageous decks, respectively. The results showed a main effect of type of deck, *F*(1, 32) = 4.802, *p* < 0.05, *η*^*2*^_*p*_ = 0.130, a main effect of region, *F*(2, 64) = 16.250, *p* < 0.01, *η*^*2*^_*p*_ = 0.337, and a main effect of group, *F*(1, 32) = 5.159, *p* < 0.05, *η*^*2*^_*p*_ = 0.139. The main effect of laterality was not significant, *F*(2, 64) = 0.594, *p* > 0.05, *η*^*2*^_*p*_ = 0.018.

The four-way interaction was not significant (*p* > 0.05); only a three-way interaction between the type of deck, region and group was found, *F*_(2, 64)_ = 4.499, *p* = 0.034, *η*^*2*^_*p*_ = 0.123. Further analysis of the three-way interaction revealed that in the high group, the P300 amplitude increased from the frontal to the parietal regions for the advantageous decks (*p* < 0.001) (see Fig 3(B)), whereas for the disadvantageous decks, the P300 amplitude of the frontal region was significantly smaller than that of the central and parietal regions (*p* = 0.005 < 0.01). In the low group, the P300 amplitude for both decks did not differ between regions (*p* > 0.05). The topographical map of p300 is in Fig 3(C).

#### Response Selection Stage

The trials completed by each participant were divided into pre-knowledge and post-knowledge stages. A 2 (group: high, low) × 2 (choice: pass, play) × 3 (region: frontal, central, parietal) × 3 (laterality: left, middle, right) mixed-design ANOVA of the mean amplitude of the DPN was completed for the two stages. For the pre-knowledge stage, a mean of 75 (SD = 4.5) and 117 (SD = 3.2) artifact-free trials were used in the analysis for the pass and play categories, respectively. The analysis of the DPN (-800 ms to 0 ms before the response) showed a significant main effect of group, *F*_(1, 32)_ = 4.324, *p* < 0.05, *η*_*p*_^*2*^ = 0.119. A significant two-way interaction between choice and group was also found, *F*_(1, 32)_ = 5.626, *p* < 0.05, *η*_*p*_^*2*^ = 0.150. A simple effect analysis of the choice × group interaction showed that the effect of choice was significant only in the high group. The participants in the high group displayed greater negativity toward playing than passing (*p =* 0.024 < 0.05), whereas no difference was observed in the low group. The data of this part is in S4 File.

**Fig 4:**
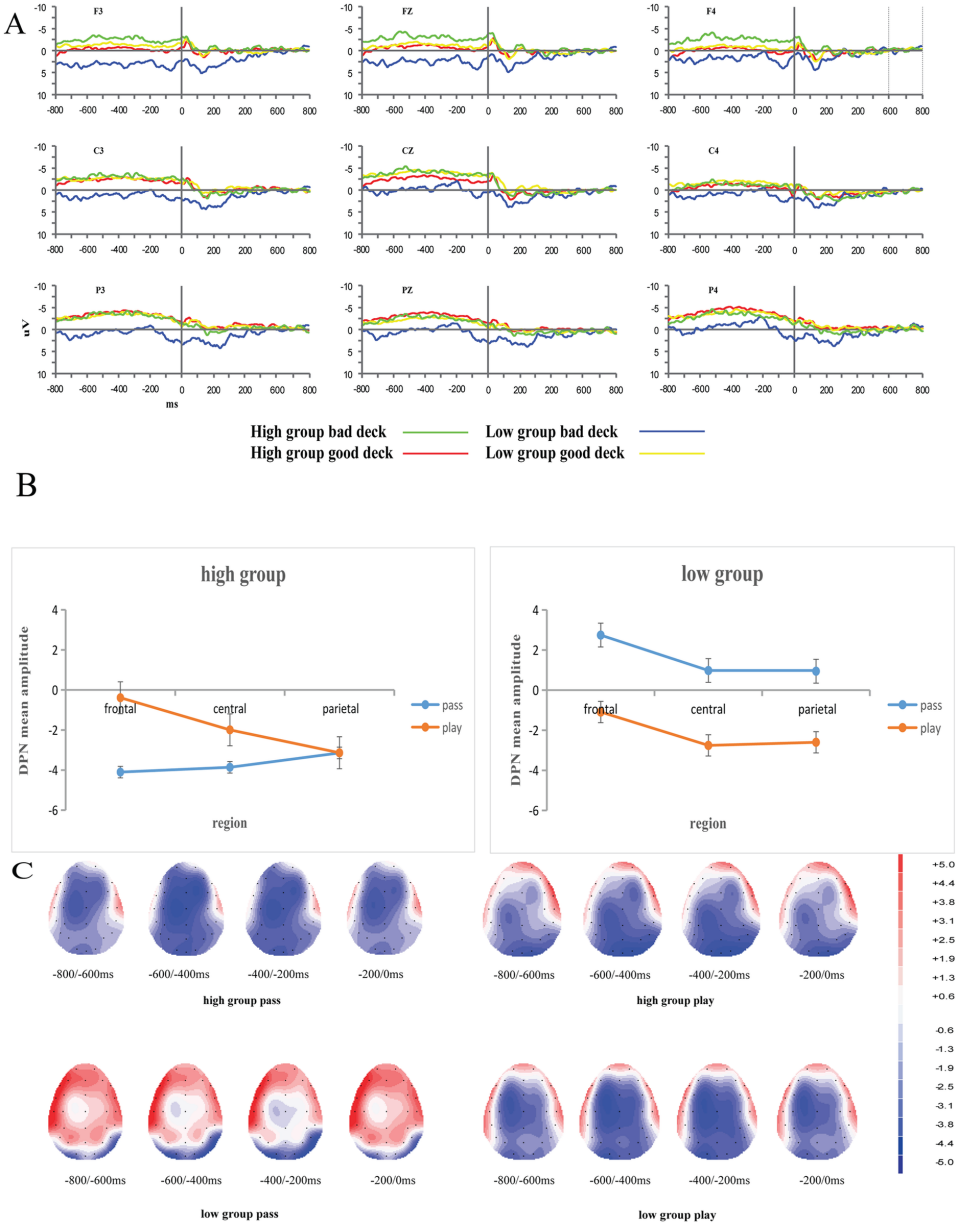
ERP results during the response selection stage. (A) Decision-preceding negativity amplitudes for different response types across groups. (B) Decision-preceding negativity amplitudes for different responses across regions. (C) Topographical maps of the difference between pass and play for the different wave forms in the two groups.

For the post-knowledge stage, a mean of 79 (SD = 3.4) and 103 (SD = 4.7) artifact-free trials were used in the analysis for the pass and play categories, respectively. Decision-preceding negativity amplitudes for different response types across groups was presented in Fig 4(A). The analysis of the first interval of DPN (-800 ms to -500 ms before response) showed a significant region main effect, *F*_(2, 64)_ = 4.546, *p* < 0.05, *η*_*p*_^*2*^ = 0.124, indicating that the mean amplitude in the central and parietal regions was more negative than that in the frontal region. The main effects of choice, laterality and group were not significant. The four-way interaction was not significant (*p* > 0.05), although a significant three-way interaction between choice, group and region was found, *F*_(2, 64)_ = 8.691, *p* < 0.01, *η*_*p*_^*2*^ = 0.214. A simple effect analysis of the choice × region × group interaction showed that the effect of choice on the frontal region was significant in both groups. The participants in the high group displayed greater negativity toward passing (*p =* 0.021 < 0.05), whereas those in the low group displayed greater negativity toward playing (*p =* 0.017 < 0.05). The effect of choice on the central (*p =* 0.018 < 0.05) and parietal (*p =* 0.012 < 0.05) regions was significant in the low group (see Fig 4(B)). These participants displayed greater negativity toward playing.

The second interval analysis (-500-0 ms before response) was the same as the first interval. There was a significant region main effect *F*_(2, 64)_ = 6.550, *p* < 0.01,*η*_*p*_^*2*^ = 0.170. This result indicates that the mean amplitude in the central and parietal regions is more negative than in the frontal region. The main effect of choice, laterality and group was not significant. The four-way interaction was not significant (*p*>0.05), and a significant three-way interaction between choice, group and region was found, *F*_(2, 64)_ = 10.432, *p* <0.01, *η*_*p*_^*2*^ = 0.246. A simple effect analysis of the choice × region × group interaction showed that the effect of choice was significant in the frontal region in both groups. The participants had greater negativity toward passing in the high group (*p =* 0.028 < 0.05) and greater negativity toward playing in the low group (*p =* 0.018 < 0.05). In the central (*p =* 0.026 < 0.05) and parietal (*p =* 0.017 < 0.05) regions, the effect of type of choice was significant in the low group. Participants had greater negativity toward playing. The topographical map of DPN is in Fig 4(C).

#### Feedback Evaluation Stage

Analyses of the feedback-related component (FRN) were conducted for all stimuli except for those trials in which the participants selected to pass because learning about contingency was not relevant for this analysis. The data of this part is in S5 File.

**Fig 5:**
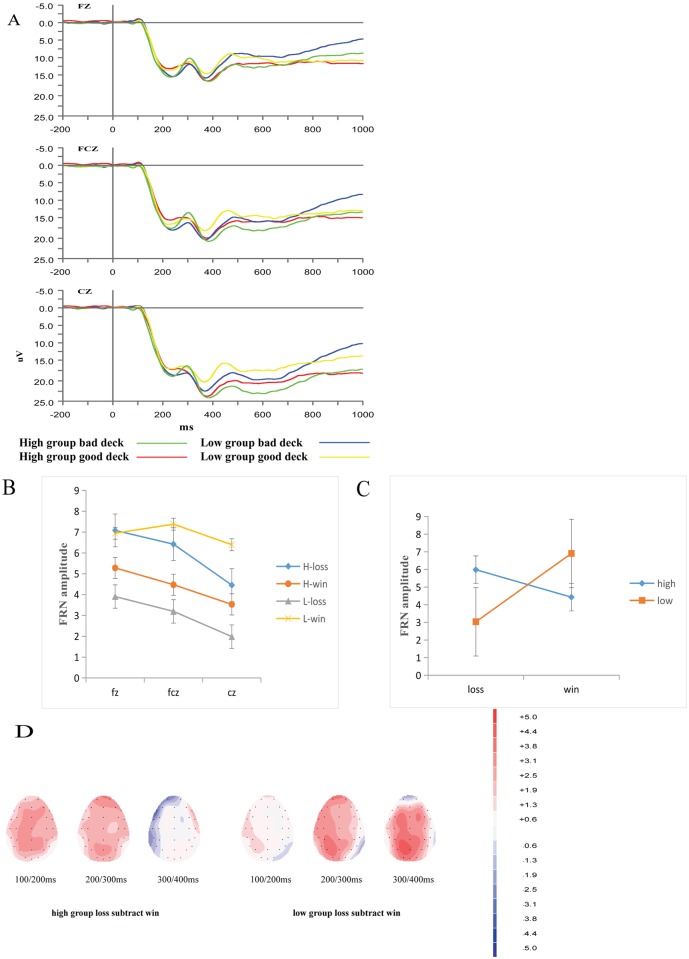
ERP results during the feedback evaluation stage. (A) The effects of valence on FRN amplitude across groups. (B) FRN amplitude on three electrodes for different valences and groups. (C) The effects of group and valence on FRN amplitude. (D) Topographical maps of the difference between loss and win (loss subtract win) for the different wave forms in the two groups.

A 2 (valence: win, loss) × 2 (group: high, low) × 3 (electrode: FZ, FCZ, CZ) mixed-design ANOVA of FRN amplitude was conducted. The effects of valence on FRN amplitude across groups was presented in Fig 5(A). A mean of 162 (SD = 3.4) and 96 (SD = 3.9) artifact-free trials were used in the analysis for the win and loss categories, respectively. Consistent with prior studies, the results showed that the FRN was sensitive to valence. The main effect of electrode was significant, *F*_(2, 64)_ = 15.038, *p* < 0.01, *η*_*p*_^*2*^ = 0.320, indicating that the FRN amplitude for loss was largest on the FZ, intermediate on the FCZ, and smallest on the CZ (see Fig 5(B)). Furthermore, the main effect of group was significant, *F*_(1, 32)_ = 5.647, *p* < 0.05, *η*_*p*_^*2*^ = 0.150. In the loss condition, the high group participants had a larger FRN amplitude than the low group participants. The main effect of valence was not significant, *F*_(1, 32)_ = 3.761, *p* > 0.05, *η*_*p*_^*2*^ = 0.105.

The three-way interaction was not significant (*p* > 0.05), but there was a significant interaction between valence and group, *F*_(1, 32)_ = 7.088, *p* < 0.05, *η*_*p*_^*2*^ = 0.181. The interaction indicated that in the high group, losses evoked a larger FRN than gains (*p =* 0.001 < 0.01). The opposite result was found in the low group (*p =* 0.045 < 0.05) (see Fig 5(C)). The topographical map of FRN is in Fig 5(D).

#### Correlation Analysis of WCST Performance and ERP Components

To mechanistically explain the connection between cognitive flexibility and decision making, we analyzed the correlation between the WCST performance and the P300 (amplitude difference between bad and good decks), DPN (amplitude difference between pass and play) and FRN (amplitude difference between loss and win) effects. The data of this part is in S6 File. Regarding the WCST performance criterion, the number of categories completed indicates the number of accurate classifications (range from 0 to 6; the completion of 6 categories reflects good cognitive function), and the number of trials administered indicates the number of trials required to learn the rules (range from 60 to 128; the completion of 128 trials reflects poor cognitive function); both of these measures thus reflect cognitive function. Every member of the high group completed 6 categories, so we could not use the number of categories completed for the correlation analysis of the high group. Similarly, every member of the low group completed 128 trials, so the number of trials administered could not be used in the correlation analysis of the low group. However, the number of categories completed was negatively correlated with the number of trials administered, consistent with the fact that they both reflect cognitive function, r = −0.948, p < 0.01. Therefore, in this study, the number of categories completed was used as a measure of cognitive function in the low group, and the number of trials administered was used as a measure of cognitive function in the high group. The percentage of correct responses indicates the percentage of correct responses out of the total number of responses and reflects the ability for abstract generalization. The number of perseverative errors indicates the degree to which the participant adhered to the old rule once the rule had changed and reflects concept formation and set shifting. Learning to learn, which reflects the ability to utilize previous experience effectively, was assessed as the average difference in the percentage of wrong responses between two adjacent classification stages.

The results showed that the relationship between WCST performance and the cognitive process underlying IGT performance differed between the two groups of participants (see Table 3). The number of trials administered during the WCST was negatively correlated with the amplitude of P300 and that learning to learn was positively correlated with the P300 in the high group. The high group’s ability to distinguish between bad and good decks is consistent with experiential learning and their cognitive ability. However, in the low group, the number of categories completed and the percent correct responses were negatively correlated with the P300, indicating that cognitive ability and abstract generalization ability were negatively correlated with discrimination between the decks in the low group. The DPN was not related with the cognitive process of the WCST in either group. The number of perseverative errors was negatively correlated with the FRN in the high group, suggesting that the abilities of set shifting and concept formation are related with the process of feedback learning. However, the FRN in the low group was negatively correlated with the P300.

**Table 3:** Correlation analysis of WCST performance and ERP components.

	P300 (CZ)		DPN (FZ)		FRN (FZ)	
	High group	Low group	High group	Low group	High group	Low group
Categories completed		-.609**		-.135		.483
Trials administered	-.629**		0.204		-0.105	
Percent correct responses	-.427	-.633**	-.141	-.178	.005	.507
Perseverative errors	-.002	-.242	.380	.069	-.506*	.390
Learning to learn	.985**	-.220	-.259	.113	-.003	.319
P300	1	1	-.329	.215	.085	-.595*
DPN	-.329	.215	1	1	-.108	.156
FRN	.085	-.595*	-.108	.156	1	1

* p < .05,

** p < .01

## Discussion

### Behavioral

The results of the present study are in line with our hypothesis that overall IGT performance would differ significantly between the high group and the low group. The high group earned more money at the end of the task. The block-by-block analysis showed that the high group demonstrated favorable decision making in block 3 as well as improvement as the IGT progressed, without any significant regression. However, the low group demonstrated unfavorable decision making for almost the entire task; although they improved by degrees, their progress was unstable (see Figs 2 and 3). The high group participants reached the conceptual knowledge stage earlier and were able to maintain favorable decision making over time. This finding might reflect the effect of WCST performance on the learning process of decision making and is in accordance with previous studies showing that WCST performance is significantly related to IGT performance.

Two parts of this study may provide insight into the reasons for the difference between the high group and the low group. First, the analysis of the subjects’ explicit knowledge of the IGT and their favorite deck showed that the high group exhibited explicit knowledge of the IGT, whereas the low group did not. Previous studies have found that IGT performance is affected by knowledge of the rules. Mellentin found that substance use disorder participants exhibited slow but stable improvement across the task [22], whereas subjects with substance use disorder and co-morbid antisocial personality disorder exhibited normal improvement initially. These differences between the groups were due to differences in the application of explicit knowledge. The second reason for the difference between the high and low groups involves the ERP results.

### ERP

This study used ERPs to explore the neural correlates of the performance of the high and low WCST groups at different stages of the IGT. The results provided sufficient evidence to explain why WCST performance is a good predictor of IGT performance.

The results for the choice evaluation stage revealed that in the high group participants, the P300 evoked in the central and parietal regions was larger than that in the frontal region when a bad deck appeared. In the low group, no differences in P300 amplitude were found between these three regions for either type of decks, consistent with our predictions. The P300, which was maximal at the centro-parietal sites among the high group participants, generally had the same amplitude across all sites among the low group participants (see Fig 3(C)). In another words, the P300 was impaired (in amplitude and latency) in the low group participants. This study did not find the same result as Cui et al. [26]; in their study, a larger P300 was observed in the left hemisphere when advantageous decks appeared, whereas a larger P300 was observed in the right hemisphere when disadvantageous decks appeared. They explained this result based on the asymmetry of cortical activity with respect to emotional function [26]. However, the P300 is not so much a symbol of emotion as a sign of attention and working memory. For example, a previous study revealed that the target-locked P300 component reflected the rehearsal and implementation of task rules in working memory [30,31]. Additionally, Horowitz-Kraus used ERP methodology to assess the WCST performance of adolescents with dyslexia and suggested that the participants’ lower performance on the WCST was due to an intact shifting mechanism but working memory deficit based on the presence of a cue-locked P300 [32]. Maurage et al. noted that if the stimulus could not be perceived well enough, the P300 could be delayed (longer latency) and less definite (reduced amplitude) [33]. The results of this study were consistent with these studies. The results of the correlation analysis revealed that the number of trials administered during the WCST was negatively correlated with the amplitude of P300 and that learning to learn was positively correlated with the P300 in the high group. The high group’s ability to distinguish between bad and good decks is consistent with experiential learning and their cognitive ability. However, in the low group, the number of categories completed and the percent correct responses were negatively correlated with the P300, indicating that cognitive ability and abstract generalization ability were negatively correlated with discrimination between the decks in the low group. Thus, the P300 deficit in the low group may have been caused by lower cognitive and abstract generalization abilities or reduced working memory ability.

In the response selection stage, the two-way interaction between choice and group during the pre-knowledge stage indicates that the responses of the low group participants did not differ between pass and play. The high group participants had stronger reactions for play. During the post-knowledge stage, the effect of choice was significant for the frontal region in both groups (*p* < 0.05). The participants in the high group displayed greater negativity toward passing, whereas those in the low group displayed greater negativity toward playing. The results for the high group participants during the post-knowledge stage are consistent with previous studies [19,26,34]. In one of these studies, the larger DPN amplitude was caused by feelings of unease and avoidance behavior [26]. Previous studies have shown that the DPN is generated by the right superior frontal gyrus, which is involved in the integration of information and potential outcomes and in the execution of motor responses and intuitive judgments [35–38]. As a slow cortical potential, the DPN reflects the regulation of local excitatory mobilization (negative slow potential) or inhibition (positive slow potential) via a threshold mechanism [39]. Those results support that the formation of somatic markers is affected by cognitive flexibility. Only participants with high cognitive flexibility can distinguish good and bad decks use somatic markers. Previous studies found the similar results that working memory and central executive affect the development of somatic markers [23–25]. Though the correlation results showed that the DPN was not related with the cognitive process of the WCST in either group. But the difference DPN reflection between pre-knowledge and post-knowledge indicate that the somatic markers are influenced by the conceptual knowledge. This results are contrary to somatic markers hypothesis which claim that somatic markers is emerge before explicit knowledge is present. Taken together, the results showed that during the pre-knowledge stage, the high group participants were more inclined to explore the rules of the decks, so they tended to “play” to identify the rules as soon as possible. These participants also acquired the correct conceptual knowledge during the post-knowledge stage. The low group participants did not adopt this strategy and their responses therefore did not differ according to their choice to play or pass. During the post-knowledge stage, the high group had identified the rules, and they formed appropriate DPN that helped the make their choices. In contrast, in the low group participants, a stronger physiological response was evoked for “play”. Because these participants continued to lack understanding of the rules through the pre-knowledge stage, they could not associate their responses with appropriate somatic markers. The difference in response patterns between the pre-knowledge and post-knowledge stages suggests that the appropriate somatic markers is formed only when participants gain conceptual knowledge. In general, the DPN reflects the process of intuitive judgment making, anticipation of the decks and affected by conceptual knowledge and cognitive flexibility. The low group participants did not show any discrimination between the decks during the pre-knowledge stage and exhibited inappropriate somatic markers and the lack of conceptual knowledge during the post-knowledge stage.

In the feedback evaluation stage, losses evoked a larger FRN than wins in the high group, but the opposite result was found in the low group. The results for the high group in the present study were consistent with previous studies [26,29,40,41], which revealed that the FRN was more sensitive to loss. According to Cui et al., the FRN reflects the process of learning from feedback [26]. Balconi argued that the FRN marks the subject’s ability to correctly match the expected outcome with the external and real outcomes [40]. Hajcak et al. also claimed that the FRN reflects the early appraisal of feedback based on binary classification of good versus bad outcomes [41]. These findings can explained by the reinforcement learning theory [42], which states that a monitoring system evaluates on-going events and evokes FRN variation through a negative reward prediction error signal that is elicited for outcomes that are “worse than expected”. The results of the correlation analysis also support the arguments described above. The number of perseverative errors was negatively correlated with the FRN in the high group, suggesting that the abilities of set shifting and concept formation are related with the process of feedback learning. However, wins evoked a larger FRN in the low group, and the FRN in the low group was negatively correlated with the P300. This result is not consistent with Cui et al. ‘s study, which found that a larger FRN effect was correlated with a better ability to discriminate between decks. This result is due to the abnormal FRN in the low group. Thus, the absence of an FRN effect in the subjects with low cognitive flexibility is due to their significant deficit in feedback learning and concept formation.

## Conclusion

This study examined two important issues, the first of which was how to define a good decision maker. The results indicated that cognitive flexibility is an effective index because the participants with high cognitive flexibility performed better on the IGT and acquired more knowledge about the task; their somatic markers were also more obvious. The second issue was the reason for the difference between two groups of participants. The IGT and WCST are both about learning about contingency, and they involve common brain areas. From the results of this study the cognitive theory is more reasonable than the somatic marker theory to explain the results. During the post-knowledge stage, the participants with high cognitive flexibility could recognize advantageous decks based on the larger P300 amplitude, had larger DPN amplitudes on “pass” choices and were more sensitive to loss when feedback was displayed. In summary, a significant finding of this study is that high cognitive flexibility can help participants learn rules quickly, and knowledge of the task reinforces the effect of somatic markers.

## Supporting Information

S1 File The data of Behavioral Results. (XLS)

S2 File The data of Knowledge of the Task. (XLS)

S3 File The data of ERP modulations in Choice Evaluation Stage. (XLS)

S4 File The data of ERP modulations in Response Selection Stage. (XLS)

S5 File The data of ERP modulations in Feedback Evaluation Stage. (XLS)

S6 File The data of Correlation Analysis between WCST Performance and ERP Components. (XLS)
